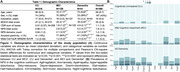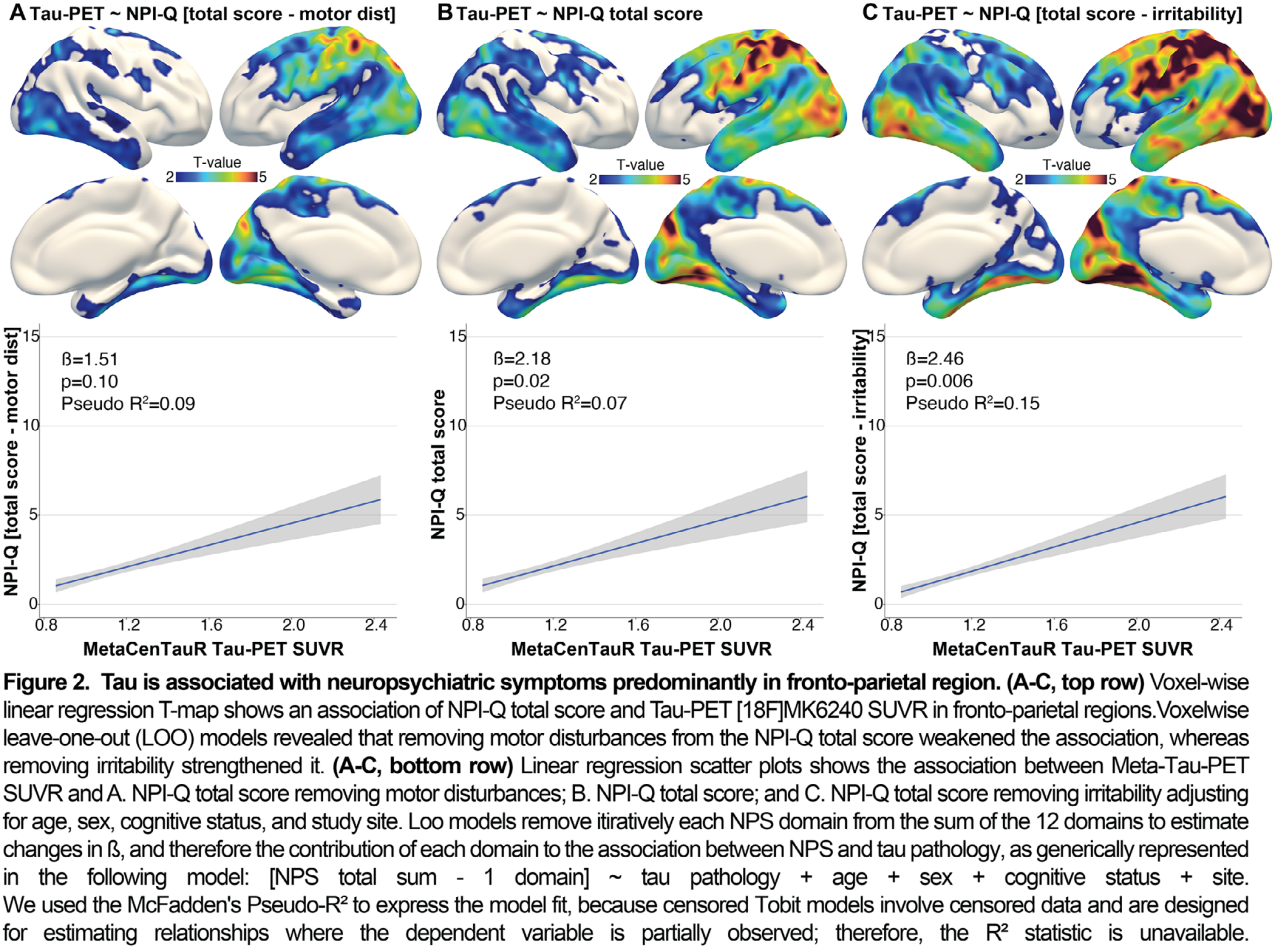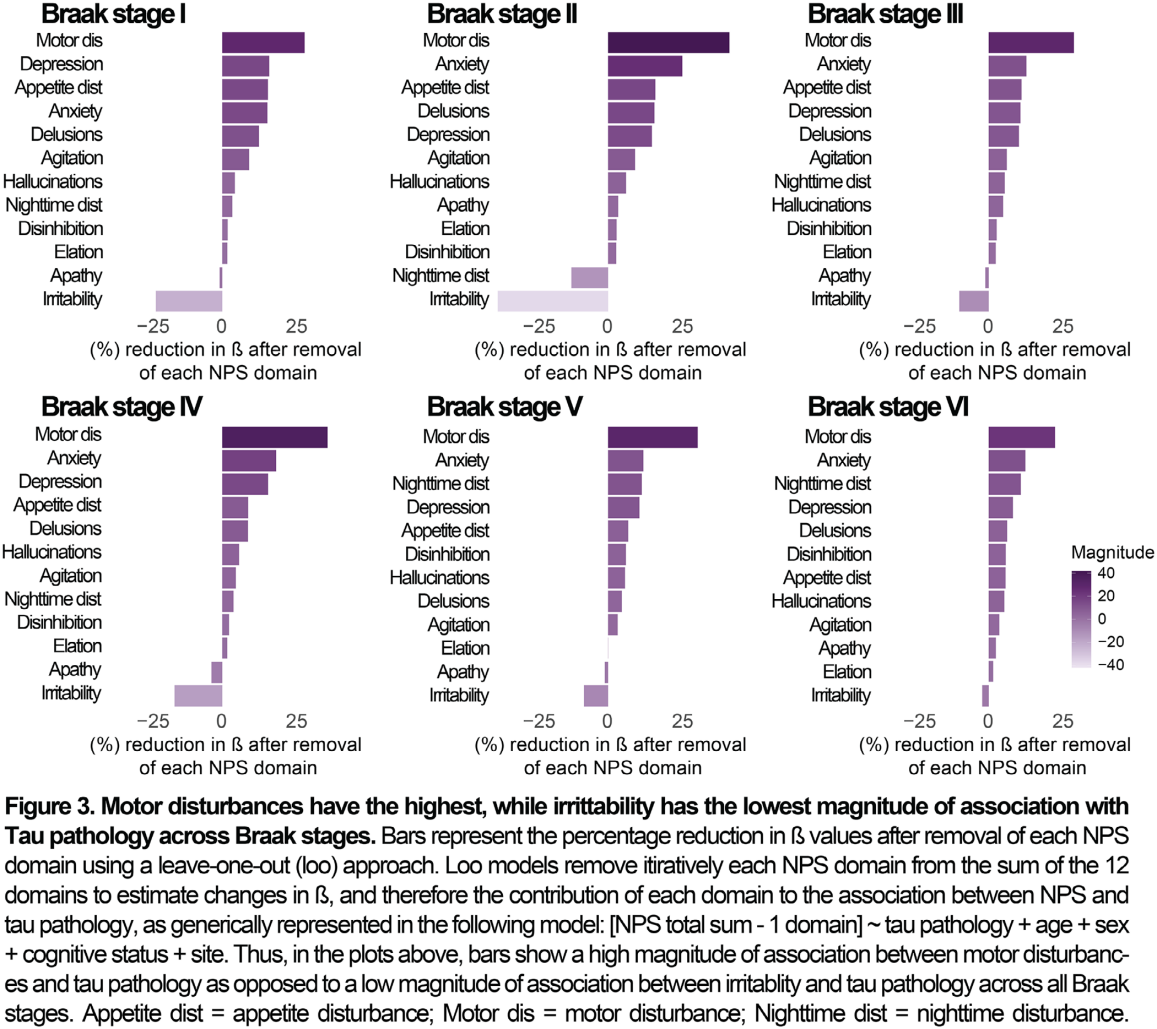# Tau exacerbates the development of motor disturbances but has no effect on the development of irritability across the Braak stages – The HEAD Study

**DOI:** 10.1002/alz70862_110260

**Published:** 2025-12-23

**Authors:** Cristiano Aguzzoli, Guilherme Povala, Pamela C.L. Ferreira, Firoza Z Lussier, Guilherme Bauer‐Negrini, Livia Amaral, Carolina Soares, Marina Scop Medeiros, João Pedro Ferrari‐Souza, Bruna Bellaver, Hussein Zalzale, Francieli Rohden, Douglas Teixeira Leffa, Lucas Porcello Schilling, Eduardo R. Zimmer, Dana L Tudorascu, Belen Pascual, Brian A. Gordon, Val J Lowe, Hwamee Oh, David N. soleimani‐Meigooni, Pedro Rosa‐Neto, Suzanne L. Baker, Tharick A Pascoal

**Affiliations:** ^1^ Brain Institute of Rio Grande do Sul (InsCer), Porto Alegre, Rio Grande do Sul Brazil; ^2^ Global Brain Health Institute (GBHI), San Francisco, CA USA; ^3^ Neurology Department, São Lucas Hospital of PUCRS, Porto Alegre, Rio Grande do Sul Brazil; ^4^ University of Pittsburgh, Pittsburgh, PA USA; ^5^ Universidade Federal do Rio Grande do Sul, Porto Alegre, RS Brazil; ^6^ Federal University of Rio Grande do Sul (UFRGS), Porto Alegre, RS Brazil; ^7^ Universidade Federal do Rio Grande do Sul, Porto Alegre, Rio Grande do Sul Brazil; ^8^ Brain Institute of Rio Grande do Sul (InsCer), PUCRS, Porto Alegre, Rio Grande do Sul Brazil; ^9^ McGill Centre for Studies in Aging, Montreal, QC Canada; ^10^ Houston Methodist Research Institute, Houston, TX USA; ^11^ Washington University in St. Louis, School of Medicine, St. Louis, MO USA; ^12^ Mayo Clinic, Rochester, MN USA; ^13^ Brown University, Providence, RI USA; ^14^ Memory and Aging Center, Weill Institute for Neurosciences, University of California San Francisco, San Francisco, CA USA; ^15^ Translational Neuroimaging Laboratory, The McGill University Research Centre for Studies in Aging, Montréal, QC Canada; ^16^ Lawrence Berkeley National Laboratory, Berkeley, CA USA

## Abstract

**Background:**

Previous studies demonstrated an association between tau pathology and the development of neuropsychiatric symptoms (NPS) in individuals with Alzheimer’s disease (AD). However, the extent to which tau influences each specific NPS domain remains unclear. Here, we aim to investigate the association of tau and each NPS domain in the AD continuum. We hypothesized that tau plays a comparatively greater effect on the emergence of hyperactive and psychotic symptoms compared to other NPS domains.

**Method:**

We assessed 385 individuals (216 cognitively unimpaired (CU), 128 MCI, and 41 AD dementia) from the HEAD study who underwent clinical assessments with the Neuropsychiatry Inventory Questionnaire (NPI‐Q) and had positron emission tomography (PET) for amyloid‐β (Aβ) ([^18^F]AZD4694 or [^11^C]PiB), and tau tangles ([^18^F]MK6240) at the same visit. Tau SUVR values were tailored with a mask from Braak stages I‐VI, using the inferior cerebellar gray matter as a reference region. All individuals with dementia had a positive Aß‐PET. Voxel‐wise and Tobit censored regression tested the association between NPS domains and biomarkers accounting for age, sex, cognitive status, and study site. We used censored regression models to account for skewed data alongside a leave‐one‐out (loo) approach to identify which NPS domains most contributed to results.

**Result:**

CI individuals had significantly higher NPI‐Q scores and Tau PET SUVR than CU (Figure 1A, 1B). NPI‐Q score was significantly associated with tau‐PET predominantly in fronto‐parietal regions. Removing irritability from the models strengthened this association (Figures 2A‐C). Regression loo models revealed that motor disturbances contributed most to the association between NPS and tau‐PET across all Braak stages. Notably, irritability had a negative effect on the association in all Braak stages, suggesting that tau does not play a role in the development of irritability and is highly associated with motor disturbances (Figure 3).

**Conclusion:**

Our study suggests that tau contributes to the development of motor disturbances but has no effect on the development of irritability across the AD continuum. These findings provide additional rationale for the development of new therapeutics aiming to mitigate motor disturbances and irritability in AD patients.